# Molecular Dynamic Simulation Search for Possible Amphiphilic Drug Discovery for Covid-19

**DOI:** 10.3390/molecules26082214

**Published:** 2021-04-12

**Authors:** Umer Daood, Divya Gopinath, Malikarjuna Rao Pichika, Kit-Kay Mak, Liang Lin Seow

**Affiliations:** 1 Division Clinical Dentistry, School of Dentistry, International Medical University Kuala Lumpur, 126, Jalan Jalil Perkasa 19, Bukit Jalil, Kuala Lumpur 57000, Malaysia; lianglin_seow@imu.edu.my; 2 Oral Diagnostics and Surgical Sciences, School of Dentistry, International Medical University Kuala Lumpur, 126, Jalan Jalil Perkasa 19, Bukit Jalil, Kuala Lumpur 57000, Malaysia; DivyaGopinath@imu.edu.my; 3 School of Pharmacy, International Medical University Kuala Lumpur, 126, Jalan Jalil Perkasa 19, Bukit Jalil, Kuala Lumpur 57000, Malaysia; mallikarjunarao_pichika@imu.edu.my (M.R.P.); kitkaymak@imu.edu.my (K.-K.M.)

**Keywords:** quaternary ammonium, amphiphile, docking, k21 spike, fit pose, Covid-19, molecular simulation

## Abstract

To determine whether quaternary ammonium (k21) binds to Severe Acute Respiratory Syndrome–Coronavirus 2 (SARS-CoV-2) spike protein via computational molecular docking simulations, the crystal structure of the SARS-CoV-2 spike receptor-binding domain complexed with ACE-2 (PDB ID: 6LZG) was downloaded from RCSB PD and prepared using Schrodinger 2019-4. The entry of SARS-CoV-2 inside humans is through lung tissues with a pH of 7.38–7.42. A two-dimensional structure of k-21 was drawn using the 2D-sketcher of Maestro 12.2 and trimmed of C18 alkyl chains from all four arms with the assumption that the core moiety k-21 was without C18. The immunogenic potential of k21/QA was conducted using the C-ImmSim server for a position-specific scoring matrix analyzing the human host immune system response. Therapeutic probability was shown using prediction models with negative and positive control drugs. Negative scores show that the binding of a quaternary ammonium compound with the spike protein’s binding site is favorable. The drug molecule has a large Root Mean Square Deviation fluctuation due to the less complex geometry of the drug molecule, which is suggestive of a profound impact on the regular geometry of a viral protein. There is high concentration of Immunoglobulin M/Immunoglobulin G, which is concomitant of virus reduction. The proposed drug formulation based on quaternary ammonium to characterize affinity to the SARS-CoV-2 spike protein using simulation and computational immunological methods has shown promising findings.

## 1. Introduction

The recent COVID-19 pandemic caused by the Severe Acute Respiratory Syndrome–Coronavirus 2 (SARS-CoV-2) that emerged in China has led to major public health concerns worldwide with more than 14.5 million infections claiming over 605,000 human lives throughout the world [1]. The SARS-CoV-2 is a member of the *Coronaviridae* family belonging to the order *Nidovirales*, which harbors the largest genome amongst all known RNA viruses, i.e., plus-stranded RNA [2]. Amongst the four generations of the coronaviruses, α-CoV and β-CoV infect mainly humans and mammals. Many subtypes have been known to cause respiratory diseases in humans; unfortunately, fatal diseases were reported amongst those cases infected by the SARS-CoV, SARS-CoV-2, and the Middle East Respiratory Syndrome Coronavirus (MERS-CoV), which belong to β-CoV. The intermediate host that transferred the SARS-CoV-2 viruses to humans has not been identified yet. A study on 1000 metagenomics samples from pangolins reported that 70% of the pangolins harbored β-CoV genome [3,4]. However, another study identified genome similarity up to 96.2% to Bat-CoV RaTG13 coronavirus detected in the bat *Rhinolophus affinis* [5].

Virus entry into host cells involves complex interactions between viral and host proteins and is a crucial determinant of infectivity. Since SARS-CoV-2 is phylogenetically closely linked to SARS-CoV, most of the findings on SARS-CoV have been utilized in elucidating the characteristics of SARS-CoV-2 [6]. In both SARS-CoV-2 and SARS-CoV, the entry process to the cell is mediated by spike surface glycoprotein that binds to the functional receptor human angiotensin-converting enzyme 2 (ACE2) [5,7]. The spike glycoprotein is composed of two subunits, S1 and S2; the S1 subunit, which specifically recognizes angiotensin-converting enzyme 2, has an N-terminal domain (NTD) and a C-terminal domain (CTD), both of which can function as a receptor-binding entity that mediates the binding of the virus to host cells [6,8]. The S2 subunit contains a membrane fusion unit, a transmembrane anchor, and a cytoplasmic tail. Usually, the spike protein assumes a trimer form with three receptor binding S1 heads on top of a trimeric membrane fusion S2 stalk [9]. For fusion with the receptor, the spike protein must undergo proteolytic cleavage at the junction of S1 and S2 to expose the fusion peptide [10,11,12].

In SARS CoV-2, the C-terminus acts as a receptor-binding domain (RBD) and region that interacts directly with ACE2 receptor-binding motif (RBM) [9,10]. It has been shown that SARS-CoV-2 shares more than 70% sequence identity of RBD with SARS-CoV [6,11]. However, the similarity in sequence identity of RBM is only 47.8% [11,13]. Although the identity of the amino acid similarity is low, the spike protein of SARS CoV-2 binds with human ACE2 in a model similar to that observed for SARS CoV [11,14]. The amino acid residue substitutions in SARS CoV-2 RBM have shown to increase the receptor binding affinity. However, despite having more affinity, SARS-CoV-2 spike protein does not bind to human ACE2 as strongly as SARS-CoV spike protein [7]. This has been attributed to the dynamic nature of RBD of the coronaviruses; the domain enables receptor binding in an upright position and does not bind to the receptors in the lying-down position [13,15]. Cryo-EM studies have illustrated SARS-CoV-2 spike protein, with RBD mostly in the “lying-down” position, with RBD in a mostly upright state in SARS-CoV spike protein [9,16]. Hence, although SARS-CoV-2 RBD has a higher receptor-binding affinity, the accessibility results in a lower binding affinity compared to SARS-CoV [12]. Moreover, this hidden RBD can evade immune cells and thus inadequate immune responses and delayed recovery.

Currently, there are no effective treatments against this novel virus, and different drugs for other microbial infections have been tried in recent clinical trials, including Remdesivir [17] (Ebola virus), Lopinavir/Ritonavir [18] (HIV), and chloroquine/hydroxychloroquine [19] (malaria). Nevertheless, the efficacy and safety of these drugs remain controversial [20]. The classical approach for novel antiviral drug discovery would be to screen compounds that inhibit the specific viral proteins playing a key role in virus–host interactions. Most of the viruses causing the zoonotic infections need to be handled in Biosafety Level (BSL) 2 or 3 facilities for in vitro experiments. Additionally, high mutation rates of RNA viruses due to their peculiar genome can lead to the selection of drug-resistant mutant phenotypes, which makes the in vitro experiments more challenging [21]. Taking into consideration the duration and cost associated involved in the experimental testing of candidate compounds in vitro, one strategy is represented by using computational modeling focusing on broad-spectrum compounds to identify novel drugs. Molecular docking studies are a promising way to explore new targets by analyzing the protein–ligand interaction or the binding free energy, which can help to predict the docked structure of a ligand–receptor complex [22]. It can also identify how certain residues can influence these protein–ligand interactions via delineating properties of proteins engaging empirical principles of physical chemistry. Viruses such as SARS-CoV-2 are classified as highly pathogenic and contagious requiring BSL 3 or 4 facilities and protocols, making drug discovery and its development an uphill task.

**Figure 1:**
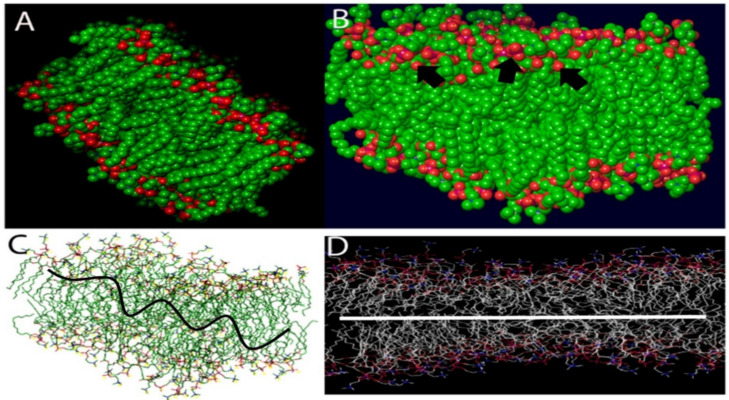
The distribution of proteins on the surface of the COVID-19 virus aligned by a random algorithm (**A**–**D**) was created with the Induced-Fit docking modules of Schrodinger 2019-4. The lipid system was generated to produce a random and organic result.

**Figure 2:**
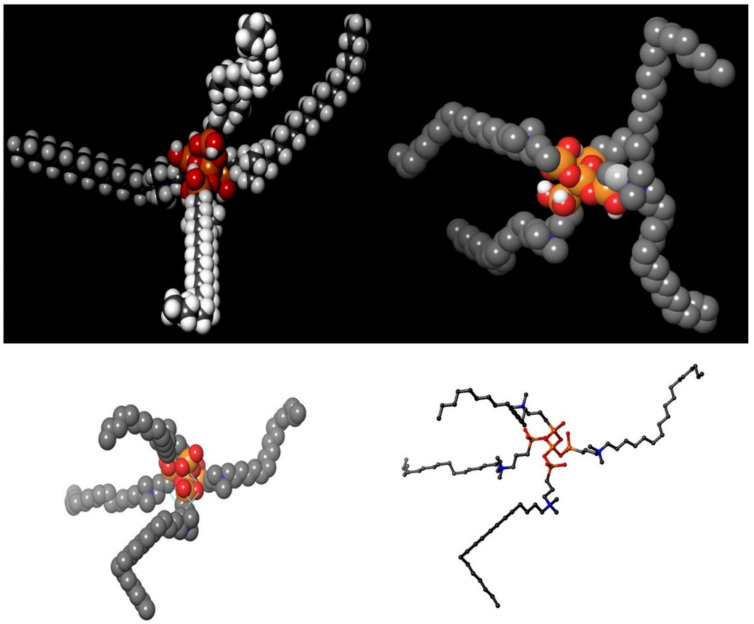
Proposed chemical formula of the quaternary ammonium molecule k21. Our formulation is based on organosilicon quaternary ammonium, 3-(trimethoxysilyl)-propyldimethy-octadecyl ammonium. It contains a long lipophilic C18 alkyl chain arm that has the ability to penetrate membranes causing cell death by direct contact and leaching of intracellular components.

Cationic amphiphilic drugs (CADs) are a broad range of chemicals having a hydrophobic aromatic ring or ring system and a hydrophilic-5 side chain containing an ionizable amine functional group [23]. Quaternary amphiphilic compounds (QACs) have both surfactant properties and broad-spectrum antimicrobial activity and hence are widely used as antibacterial agents. Although the exact mechanism behind the antimicrobial properties of QACs is not fully understood, it is well accepted that QACs can solubilize phospholipid bilayers inside membranes, leading to progressive cell lysis [24]. We can corroborate our assumption on the mode of action of the amphiphilic compounds on the viral envelope and its subsequent survival inside the host cell [25]. The inhibition of infection is independent of viral replication, with amphiphilic compounds being known to decrease the viral glycoprotein [19]. The classical approach to develop an antiviral drug is based on the compound affecting the functions of specific viral proteins that take part in the key viral life cycle. The amine functional groups present within these compounds are unprotonated within physiological pH limits, becoming protonated in an acidic environment getting trapped inside the organelles. This leads to the pronounced accumulation of drugs leading to various physiological and morphological alterations. Coronaviruses are covered by protective hydrophobic or lipid covering (Figure 1). Our proposed formulation is based on quaternary ammonium, 3-(trimethoxysilyl)-propyldimethy-octadecyl ammonium (k21), possessing potent antibacterial activities [26], and it is also used for medical devices [27]. It contains a long lipophilic C18 alkyl chain arm (Figure 2) with the ability to penetrate membranes causing cell death by direct contact and leaching of intracellular components [28]. It is speculated that our compound, named k21, has the ability to interact with this protective lipid layer of the virus and can be considered as a killer agent for such a type of virus. The present study was undertaken to determine whether k-21 binds to SARS-CoV-2 spike protein via computational molecular docking simulations. Docking studies were carried out on a crystal structure, spike receptor-binding domain complexed with hACE-2 (chain B, PDB ID: 6LZG). Glide (Standard precision (SP) mode and Extra precision (XP) mode) and induced-fit docking modules of Schrodinger 2019-4 were used to evaluate the interactions between K-21 and the spike receptor-binding domain. From the binding energy and interaction studies, K-21 showed the affinity toward the spike receptor-binding domain; thus, it can prevent its binding with ACE-2, leading to the prevention of SARS-CoV-2 penetration into humans. The conventional method of drug development, involving entire organisms or large proteins, leads to unnecessary antigenic load along with increased chances of allergenic responses. This problem can be overcome by developing drugs comprising short immunogenicity with the ability to elicit strong and targeted immune responses [29]. Recent advancements in computational simulation biology have opened up new doors for designing effective vaccines in silico [30,31]. In this study, the in silico approach has been applied for attaining a drug against Covid-19 that induces the activation of immune cells.

## 2. Results

The distribution of proteins on the surface of the SARS-CoV-2 virus was aligned by a random algorithm (Figure 1A–D) and created with the Induced-Fit docking modules of Schrodinger 2019-4. The lipid layer was generated to produce a random and organic result. The proposed chemical formula of the quaternary ammonium molecule k21 is shown in Figure 2. Our proposed formulation is based on organosilicon quaternary ammonium, 3-(trimethoxysilyl)-propyldimethy-octadecyl ammonium. It contains a long lipophilic C18 alkyl chain arm that can penetrate membranes causing cell death by direct contact and the leaching of intracellular components.

**Figure 3:**
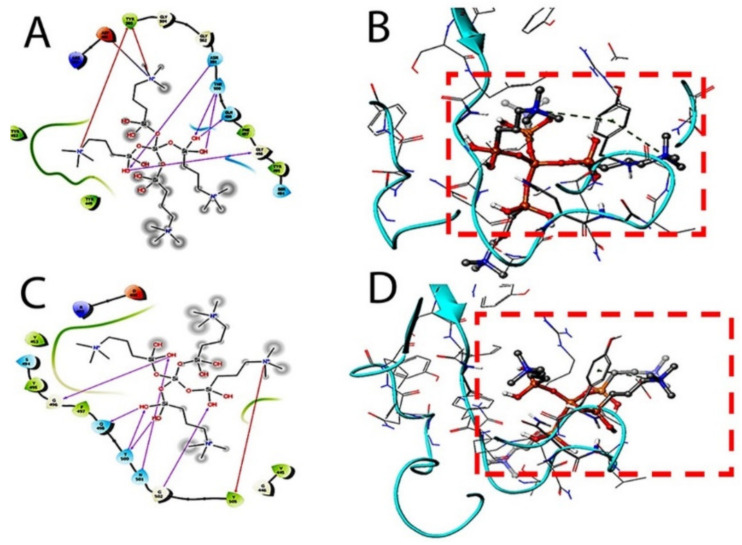
Docking studies on k-21 with spike protein (PDB ID: 6LZG, Chain B). The protein and ligand preparation were done using a force field—OPLS3e at a pH of 7.0 ± 2 with default settings. The Glide module was used to dock the quaternary ammonium compound into the binding site grid using Standard precision (SP) 2D (**A**) and 3D (**B**) with Extra precision (XP) docking 2D (**C**) and 3D (**D**) protocols with default settings. In SP and XP docking protocols, the ligand is made flexible, and the receptor was made rigid.

The therapeutic probability against SARS-CoV-2 was established using prediction models with negative and positive control drugs (data not shown). The detailed observations of docking poses of quaternary ammonium compound and its interactions with key residues of the binding site in all the three docking protocols (SP, XP, and Induced-Fit) revealed that the interactions are consistent with reasonable docking and induced-fit docking (IFD) scores. The negative scores indicate that binding of the quaternary ammonium compound with spike protein’s binding site is favorable. The two-dimensional (2D) and three-dimensional (3D) interaction diagrams of the quaternary ammonium compound within the binding site, the docking scores, IFD scores, the receptor residues involved in binding, and the nature of the interaction (hydrogen bonding, hydrophobic bonding, etc.) was shown in Figure 3. Here, we defined the docking position of the sites relevant for protein function, which is the interaction site of the spike protein identifying the amino acid residues involved in drug binding. The results are best shown in Table 1 and the drug-induced poses.

**Figure 4:**
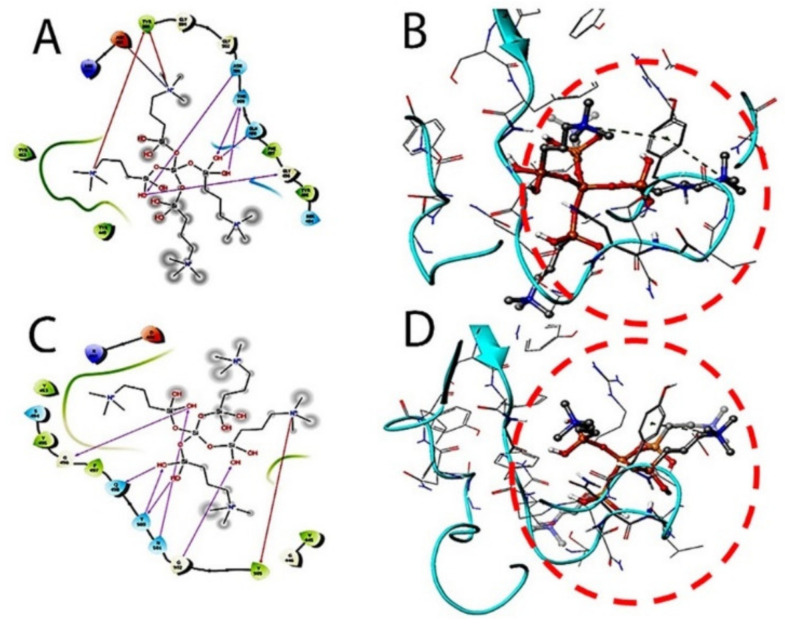
The induced fit pose 1 (**A**,**B**) docking active ligand with Glide using van der Waals removing side chains during the k21/quaternary ammonium silane docking step. The induced fit pose 2 (**C**/2D, **D**/3D) was used to accommodate the ligand by reorientation of the side chains with low energy proteins ranked according to Glide Score.

**Figure 5:**
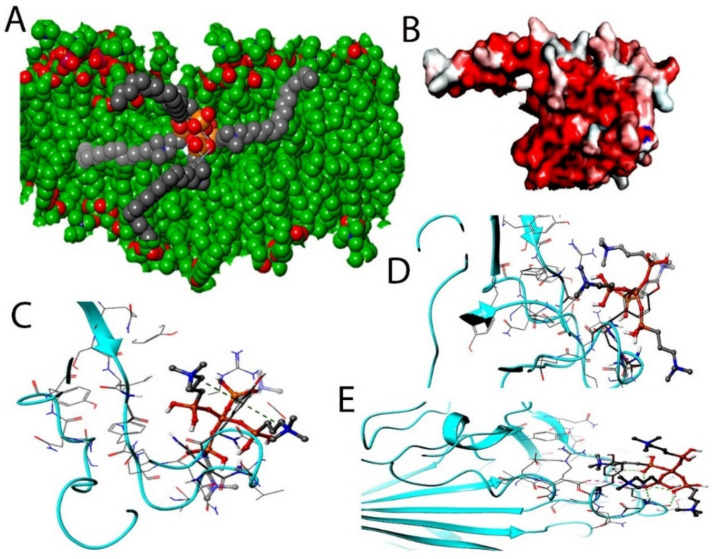
(**A**) COVID-19 bilayer final structure snapshot with k21. (**B**) The spike interaction site with ACE-2 (**C**,**D**). The induced fit pose 4 (**A**,**B**) docking active ligand with Glide during the k21/QA docking step. (**E**) The induced fit pose 5 according to Glide Score.

The target interactions with the spike proteins were highest for the drug formulation. The protein–drug interactions are illustrated in Figure 3, Figure 4 and Figure 5.

**Figure 6:**
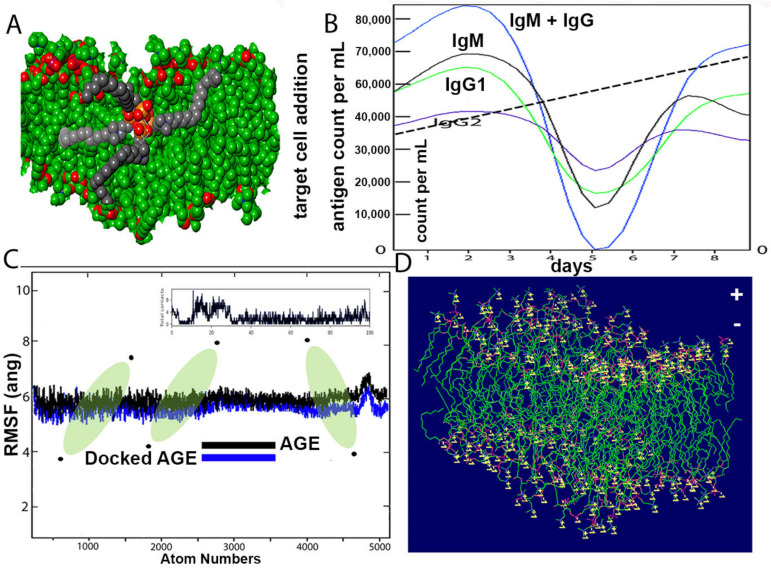
(**A**) Structure of COVID-19 wall and k21/QA formulation after equilibration. (**B**) Computational immune stimulation of the host immune system response to k21/QA with predicted concentration densities. The IgG and IgM concentrations against the formulation are shown. Each color represents measurements against the amphiphile drug monitored for up to 8 days. The total number of antigen count per microliter was measured using an automated cell counter with increased viral fitness within the set parameters represented by a dashed line also signifying experimental effects are equal. (**C**) Root Mean Square Fluctuations of the COVID-19 structure and its docked form with k21/QA. (**D**) Illustration of a typical viral wall structure. The wall is simulated for the amphipathic drug anchored to a lipid bilayer by terminal positively charged residues (lysine or arginine). Conformational changes in the structure regulate the flow of ions through the porous wall.

The structure of the SARS-CoV-2 wall and k21/QA formulation is seen after equilibration (Figure 6A). The Root Mean Square Deviation fluctuation with simulation is seen in Figure 6C. Within a span of 2 nanoseconds, there is considerable interaction between the proposed drug and ACE spike proteins. The docking protein calculated a distance of 2.3 Å between the drug molecule and ACE. The drug molecule has a large RMSD fluctuation due to the less complex geometry of the drug molecule, which is suggestive of a profound impact on the regular geometry of the viral protein. The immune response, both primary and secondary, play a significant role against the virus (Figure 6B). There is a high concentration of IgM and IgG at all stages, which is concomitant to virus reduction. All these suggest the efficient immune response of the drug and clearance of the pathogen upon simulated encounters. The immunological memory is developed during the first response and is much more rapid, which shows the time the immune system takes to clear the antigen. In summary, the dynamics are consistent with a realistic immunization process, because they show a faster response due to the development of long-lasting memory. The dipole–dipole attractive forces formed when hydrogen atoms bonded to the electronegative atoms of the drug simulated rigidity in achieving a stable conformation with a cut-off distance of 3.0 Å. The maximum number of hydrogen bonds between the drug formulation and the virus is 12. Figure 6D indicates the typical viral wall structure simulated for the amphipathic drug anchoring on the lipid layer. The wall is seen with positively charged residues (lysine or arginine), as conformational changes in the structure regulate the flow of ions through the porous wall. Binding free energies are comprised in the range 4–9 kcal/mol and are found to be strongly correlated. Most of the residues bear 2 to 5 H-bond acceptor or donors.

## 3. Discussion

Receptor binding of ACE2 and the viral S protein is mediated by the proteaseTMPRSS2 1 [32,33]. Subsequently, the viral S protein interacts with the extracellular domain of ACE2, which activates clathrin-dependent endocytosis of the whole complex [32,34]. However, once the SARS-CoV-2 infects human cells, unknown viral mediators are thought to inhibit ACE2 and parallelly induce the expression of certain metalloproteases involved in releasing the tumor necrosis factor α (TNFα) and other pro-inflammatory cytokines such as interleukin 4 (IL-4) and interferon γ (IFNγ) [35,36]. The latter two have been known to downregulate ACE2 expression on the cellular surface of infected cells via autocrine pathways [37,38]. Once the virus gains entry to cells, the RNA gets released and it uses host-cell machinery for viral replication. The S-protein is known to prompt the fusion of endosomal and viral membranes, releasing the viral genome into the cytoplasm. Cationic amphiphilic drugs have also been known to interact with cell membranes and accumulate in acidic intracellular compartments of lysosomes and endosomes [39]. The cellular uptake mechanisms and accumulation depend on the physicochemical properties of the molecules [40,41]. At physiological pH, the amine group of these compounds is mainly unprotonated. Once internalized by cellular components with an acidic environment, the groups become protonated and cannot permeate the membrane [39,42].

As evident from Figure 3, Figure 4 and Figure 5, the SARS-CoV-2 virus shows considerable conformational changes due to the translational and rotational movement of the drug molecule over the virus structure. The docked protease structure shows a profound impact on the regular geometry of the AGE as a result of the drug interaction. Table 1 represents docking scores, docked structures, and fit poses of the k21/QA drug formulation used for screening with COVID-19. The negative scores indicate that the binding of quaternary ammonium compound within spike protein’s binding site is favorable. The number of H-bonds has increased, and as the drug enters the spike protein’s binding site, it forms additional H-bonds even though the average distance of H-bond increases.

The simulations of solvated bilayers were performed for comparison purposes. The purpose was not to accurately describe the lipid bilayer systems, because the focus lies on the relative drug effect, and therefore, the discussion does not hamper our conclusions. This interaction between the bilayer and the drug does not appear to be due to a single amino acid but to a wide set of amino acid residues, promoting a more stable adsorption/interaction of the drug with the bilayer.

In addition, the primary and secondary immune responses may play a pivotal contribution against the pathogen. [43]. The in silico immune response is shown in Figure 6B. The high concentrations of IgM and IgM + IgG suggest an efficient response and clearance of the pathogen upon subsequent encounters with the drug. Molecular simulations have been used to assist the discovery and development of antiviral drugs, which show sampling snapshots of drug interactions and fluctuated protein structures [44].

3-(trimethoxysilyl) propyldimethyl octadecyl ammonium is a SiQAC ((3-(trimethoxysilyl) Propyldimethyl Octadecyl Ammonium Chloride)) by Dow Corning and possesses mild antiviral activity against influenza A virus [45]. Ethoxylated SiQAC with three additional molecules of 3-(triethoxysilyl)propyldimethyl octadecyl created a k21/QA molecule having four quaternary arms imparting four positive charges to the molecules conferring a strong and robust antiviral property. The k21 molecule can directly affect the virus, altering the infectious properties with significant altering of the attachment and entry of SARS-CoV-2. When directly exposed to the virus, it inhibits viral survival, altering viral attachment. It is speculated that the k21 might also change the cell physiology, making it non-conducive for viral replication. Hence, the ability of k21 to regulate SARS-CoV-2 and inhibit its spread makes it a potentially effective antiviral compound. Furthermore, engineering animal models to study these drugs will allow the selection of new classes of molecule, with innovative technologies bringing more breakthroughs to these formulations. Further in-depth studies will be essential in order to understand the exact mode of action of this proposed formulation.

The molecular dynamics (MD) simulations were not performed, as the authors had confirmed the binding through glide docking (both SP & XP) studies, induced-fit docking studies, and binding energy calculations. In addition, the membrane permeability studies were also not considered, mainly because the spike protein is present on the outside of the membrane. So, it is an understanding that membrane permeability studies may not be necessary and required for the objective of the present study. However, in our previous studies, we have reported the antibacterial effect of k21 [24], suggesting that the compound k21 can permeate through the membranes.

The PDB ID of the SARS-COV-2 spike protein crystal structure used in this study is 6LZG. This crystal structure is of resolution 2.50 Ǻ, in which the interaction between spike (s) protein of SARS-CoV-2 and human angiotensin-converting enzyme 2 (hACE2) were shown. The key amino acid residues involved in the interaction are A475, E384, Y453, K417, G446, Y449, G496, Q498, T500, G502, Y489, and F486. Thus, the binding site (receptor-grid) with these amino acids as the centroid was generated using the “grid receptor generation” module with default settings. The lowest energy conformation of k21 was docked into the binding site using the Glide module with Standard precision (SP) and Extra precision (XP) scoring functions [46]. The SP docking score was found to be −3.471 Kcal/mole, and the XP docking score was found to be −5.359 Kcal/mole. In SP docking protocol, k21 formed H-bond interactions with G496, T500, N501, and G502. In XP docking protocol, k21 formed H-bond interactions with G496, T500, and N501. The formation of an H-bonding interaction between k21 and spike protein indicates that their binding interaction is stable. In the glide docking protocol, only the ligand (k21) was made flexible, while the receptor (S protein) was made rigid.

However, in a physiological environment, the ligand and receptor are expected to be flexible. Thus, we have performed the induced-fit docking studies [47] to confirm the stability of the interactions between k21 and the amino acid residues in the binding pocking to spike protein. In induced-fit docking (IFD) protocol, the receptor around the ligand was also made flexible and thus somehow to mimic the physiological environment. Thus, in this study, the binding of k21 with S protein was further confirmed by performing induced-fit docking studies following the “induced fit docking module”. In induced-fit docking, the amino acids present in the 5 Ǻ radius around the ligand were made flexible. In all the poses generated from IFD, the H-bond interactions found in SP and XP docking remained unaffected and thus indicating the stable interaction between k21 and spike protein. The binding energy of the k21/spike protein complex was calculated using “Prime MM-GBSA protocol” with default settings in Schrodinger’s Drug Discovery Suite. The binding energy (∆G) was found to be −71.74 Kcal/mole. The results from molecular docking studies in SP and XP protocols, induced-fit docking studies, and binding energy calculations are in agreement, with each suggesting the interaction between k21 and the binding pocking of spike protein and suggesting that the interaction is thermodynamically favorable and stable.

## 4. Materials and Methods

### 4.1. Protein Preparation

The crystal structure of the SARS-CoV-2 spike receptor-binding domain complexed with ACE-2 (PDB ID: 6LZG) was downloaded from RCSB PDB/. It was prepared for molecular docking studies using the protein preparation wizard of Schrodinger 2019-4 (Schrödinger Release 2021-1: BioLuminate, Schrödinger, LLC, New York, NY, USA) using an OPLS-3e force field at pH 7.40 ± 0.02 and the other default settings. The entry of SARS-CoV-2 inside humans is through lung tissues with a pH of 7.38–7.42; therefore, the protein was prepared at pH 7.40 ± 0.02. The SARS-CoV-2 spike protein binds with ACE2 of the lungs. In the crystal structure, 6LZG, chain A is hACE-2 protein and chain B is spike protein. From the prepared protein complex of 6LZG, the chain B was extracted and used for further docking studies.

### 4.2. Binding Site Detection and Preparation

The amino acids of spike protein involved in its binding with ACE-2 of human lungs have been reported by Wang et al. [48]. Strong polar interactions were reported between A475 of spike protein A474 and hACE-2 S19, N487 with Q24, spike protein E484 with hACE-2 K31, and spike protein Y453 with hACE-2 H34. Spike protein K417 forms ionic interactions with hACE2 D30. Spike protein residues (G446, Y449, G496, Q498, T500, and G502) interact with hACE-2 D38, Y41, Q42, K353, and D355 through H-bonds. In addition, spike proteins Y489 and F486 form hydrophobic interactions with hACE2 F28, L79, M82, and Y83. Thus, the binding site of spike protein with ACE-2 was defined using the spike protein’s amino acid residues (E384, Y453, G446, Y449, A475, F486, Y489, G496, Q498, T500, and G502) as the centroid of the cavity, and the receptor-binding site was generated using the receptor grid generation module of Schrodinger 2019-4 with an OPLS-3e force field and default settings.

### 4.3. Quaternary Ammonium Compound Preparation

The two-dimensional structure of K-21 was drawn using a 2D-sketcher of Maestro 12.2 (Maestro, version 9.3.5. Schrödinger, LLC; New York, NY, USA). K-21 is a very big molecule with central silanol moiety attached to 4 arms of C18 substituted tertiary amino silanols. Thus, the whole structure cannot be used as a ligand. In this study, we have trimmed the C18 alkyl chain from all four arms and performed molecular docking studies with the assumption that the core moiety K-21 without C18 groups is the major determinant of binding interactions within the binding site. The quaternary ammonium silanol compound was minimized and prepared for molecular docking studies using the “Ligand preparation” module with the default setting at pH 7.40 ± 0.02 and an OPLS3e force field.

### 4.4. Molecular Docking Studies and Initial Poses Generation

A glide module was used to dock the quaternary ammonium compound into the binding site grid using Standard precision (SP) and Extra precision (XP) docking protocols with default settings. In SP and XP docking protocols, the ligand is made flexible, and the receptor was made rigid. To further confirm the binding efficacy of the quaternary ammonium compound, induced-fit docking studies were performed using the “induced-fit docking” module. In this, the ligand was made flexible, and all the residues within the range of 5 Å of the receptor were made flexible. In general, induced-fit docking provides better insights into binding interactions and efficacy. The poses with the highest negative docking scores are shown in the results.

### 4.5. Immune Profiling of k21/QA

The immunogenic potential of k21/QA was conducted using the C-ImmSim server (http://150.146.2.1/C-IMMSIM/index.php accessed on 7 November 2020) for position-specific scoring matrix analyzing the human host immune system response. The system consists of cellular automation with IgM and IgG of all stages concomitant of virus reduction. The immune system input parameters were set for simulation using random seed (12345), volume (10), number of steps (100), and number of drugs instils, which was set to 1. The remaining parameters were set as default with consideration of immune system response from three sites of lymph nodes, thymus, and bone marrow as tertiary sites, keeping mammalian immune response against the drug construct the pivotal point. During each time set up, the interactions amongst cells, molecules, and the drug took place on a lattice site. The model represented IgM and IgG as “active” or “resting” taking no native cells into account. The C-ImmSim server incorporated antigen processing and presentation. The simulation as mentioned with default parameters included steps set at 1000 (random seed ¼ with the input of k21/QA drug-containing LPS (lipopolysaccharide) wall. All entities were set with different phases displaying recognition and response processes of the immune system against the virus. In this experiment, we test the emergence of one or more dominating stages of chronic exposure to the same immunogenic molecule. In other words, we check if the system reproduces the phenomenon of affinity maturation. To mimic chronic exposure to the virus, we repeatedly introduce a certain amount of ACE-2 throughout the simulation period. The system responds by mounting a specific immune response from the beginning of the exposure. This is shown in the result in Figure 6B.

### 4.6. MD Simulations: Protein–k21/QA Formulations

Minimum energy configurations were obtained from the docking studies using a protein preparation wizard of Schrodinger 2019-4 using an OPLS-3e force field along with the CHARMM36-mar2019 force-field. The ligand parameters were generated using the CHARMM General Force Field server between two successive images of the complex using periodic boundary conditions using isochoricisothermal (NVT) equilibration at 300 K.

## 5. Conclusions

The proposed drug formulation based on organosilicon quaternary ammonium, 3-(trimethoxysilyl)-propyldimethy-octadecyl ammonium, to characterize affinity to the SARS-CoV-2 spike protein using simulation and computational immunological methods has shown promising findings. The k21 formulation can provide a direction suitable for drug development to bring a possible treatment modality to the COVID-19 pandemic.

## Figures and Tables

**Table 1:** Docking scores, docked structures, and fit poses of the k21/QA drug formulation used for screening with COVID-19.

Docking Score	−3.471 Kcal/mole	−5.359 Kcal/mole	−425.46 Kcal/mole	−425.09 Kcal/mole
	H-Bonding			
Amino Acid	Distance (Å)	Distance (Å)	Distance (Å)	Distance (Å)
G496 (HA)	1.85	1.85	1.60	2.23
Q498 (HA)			1.75	1.79
T500 (HA)	1.84	1.94	1.57	2.07
T500 (HD)	1.76		1.95	1.94
N501 (HD)			1.68	1.85
N501 (HA)	1.89	2.27		
G502 (HD)	2.02			2.18
N501 (HD)		2.12		
Hydrophobic interactions	Y453, F497, Y495, Y505	Y453, Y495, V503, Y505	Y449, Y453, Y495, G496, F497, Y505	V445, Y453, Y495, F497, Y505
G Score			−5.721 Kcal/mole	−5.087 Kcal/mole
XP G Score			−5.359 Kcal/mole	−5.359 Kcal/mole
Y505			Pi-cation interaction	Pi-cation interaction
D405			Salt bridge interaction	

## Data Availability

The data can be provided by authors on reasonable request.

## Sample Availability

Samples of the compounds are not available from the authors.
